# 

**Published:** 1839-10

**Authors:** 


### BOOKS RECEIVED FOR REVIEW.

ENGLISH.

1. Tables, showing the Temperature of Cove, for the year 1838, and contrasting it with Torquay, &c. By D. H. Scott, M.D., &c. Folio, pp. 4.

2. Selections in Pathology and Surgery; or, an Exposition of the Nature and Treatment of Local Disease; exhibiting new pathological views, and pointing out an important practical improvement. Illustrated by Cases. By John Davies, Surgeon to the Hertford Infirmary.—London, 1839. 8vo, pp. 128. 6s.

3. Practical Remarks on the Use of Iodine, locally applied in various Surgical Diseases and External Injuries. Illustrated by cases. By John Davies, Surgeon to the Hertford Infirmary.—London, 1839. 8vo, pp. 62. 3s.

4. A Treatise on the Diseases of the Heart and Great Vessels, and on the Affections which may be mistaken for them, &c. By J. Hope, M.D. F.R.S. 3d Edition, corrected and greatly enlarged. —London, 1839. 8vo, pp. 639. 18s.

5. The Medical Portrait Gallery. Parts XVII, XVIII, XIX, containing Memoirs and Portraits of Jas. Ware, F.R.S., Sir Chas. Bell, F.R.S., Hippocrates, Dr. Bostock, Dr. Roget, and Mr. Stafford.—Lond. 1839. 3s. each No.

6. The Student's Guide to the Hospitals and Medical Institutions of Paris. To which is added, an outline of the Edinburgh and German Universities. By John Wiblin, M.R.C.S.— London, 1839. 12mo, pp. 90. 3s.

7. Human Physiology, for the use of Elementary Schools. By Charles A. Lee, M.D., late Professor of Mat. Med. in the University of New York. Second Edit.— New York, 1839. 8vo, pp. 336.

8. The Collected Works of Sir Humphry Davy, Bart., L.L.D., F.R.S., &c. Edited by his brother, John Davy, M.D. F.R.S. Vol. I. Memoirs of his Life.—London, 1839. 8vo, pp. 475. 10s. 6d.

9. On the Enlisting, Discharging, and Pensioning of Soldiers; with Official Documents on those branches of Duty. By H. Marshall, F.R.S.E., Deputy Inspector-General of Hospitals. Second Edition.— Edinburgh, 1839, 8vo, pp. 259. 7s. 6d.

10. The Surgical Anatomy of the Groin, the Femoral and Popliteal Regions. By Thomas Morton.—London, 1839. 8vo, pp. 207. With Plates. 9s. plain, 13s. col.

11. The Cyclopædia of Anatomy. Ed. by R. B. Todd, M.D., F.R.S. Part XVII. (Hibernation Insect.) July, 1839.

12. First Annual Report of the Registrar-General of Births, Deaths, and Marriages in England.—London, 1839. 8vo, pp. 168.

13. The Surgeon's Vade Mecum; a Handbook of the Principles and Practice of Surgery. Illustrated with numerous wood engravings. By Robt. Druitt, M.R.C.S. —Lond. 1839. Sm. 8vo, pp. 429. 8s. 6d.

14. A Practical Treatise on the Human Teeth; showing the causes of their Destruction and the means of their Preservation. By William Robertson. With Plates. Second Edition.—London, 1839. 8vo, pp. 205. 7s. 6d.

15. An Inquiry into the Morbid Effects of Deficiency of Food, chiefly with reference to their occurrence amongst the destitute poor; also, Practical Observations on the Treatment of such cases. By R. B. Howard, M.D., Physician to the Ardwick and Ancoat Dispensaries, Manchester.—London, 1839. 8vo, pp. 77.

16. Stammering practically Considered; with the Treatment in detail. By T. Bartlett, Assist. Surg. King's Own Light Infantry.—Lond. 1839. 12mo, pp. 83. 5s. 6d.

17. The Animal's Friend; or, the Progress of Humanity. No. VII. 8vo, pp. 72.—London, 1839.

18. Essays, Anatomical, Zoological, and Miscellaneous. Reprinted. By Arthur Jacob, M.D. F.R.S.A., Professor of Anatomy and Surgery in the Royal Coll. of Surgeons in Ireland.—Dubl. (no date). 8vo, pp. 126.

19. The Hunterian Oration for 1839. By Edward Stanley, Esq. F.R.S.—London, 1839. 8vo, pp. 41.

20. Nineteenth Annual Report of the Dundee Lunatic Asylum. By P. Nimmo, M.D., and A. Mackintosh, Surgeon.— Dundee, 1839. 8vo, pp. 42.

21. Inaugural Dissertation on the Physiological Inferences to be deduced from the Structure of the Nervous System in the Invertebrated Classes of Animals. (Prize Thesis.) By W. B. Carpenter.— Edinburgh, 1839, 8vo, pp. 83. 1s. 6d.

22. Affinities of Plants; with some Observations on Progressive Development. By Thomas Baskerville, Surgeon.—Lond. 1839. Small 8vo, pp. 114.

23. Retrospect of the Progress of Surgical Literature, for the year 1838-9. By Messrs. Newnham, Wickham, and Salter. Read before the Southern Branch of the Provincial Medical Association.—London, 1839. 8vo, pp. 47. 2s.

24. Observations on the Disorder of the General Health of Females, termed Chlorosis; showing the true cause to be entirely independent of Peculiarities of Sex. By Samuel Fox, Surgeon. — London, 1839. 8vo, pp. 30. 6s.

25. Cure of Club-foot, Bent-knee, Wry-neck, Spinal and other Deformities. With Remarks on the late progress of Art, and on the necessity of a Public Institution for the relief of the Poor labouring under Deformities. By Gustav Krauss, M.D.—London, 1839. 8vo.

26. Outlines of a Course of Lectures on Chemistry, delivered before the British Residents at St. Petersburg, in 1837. By George Lefevre, M.D., &c. (Not published.)—Petersburg, 1838. 8vo, pp. 152.

27. A Lecture, introductory to the Course of Instruction in the Medical Institution of Yale College, delivered Nov. 2, 1838. By Jonathan Knight, M.D., Professor of Surgery.—New Haven, 1838. 8vo, pp. 27.

28. The Annual Address to the Candidates for Degrees and Licences in Yale College, Feb. 26, 1839. By Thos. Miner, M.D.—New Haven, 1839. 8vo, pp. 20.

29. Medical Lexicon; a new Dictionary of Medical Science, containing a concise account of the various Subjects and Terms, &c. Second Edition, with numerous modifications and Additions. By Robley Dunglison, M.D. M.A.P.S., &c.—Philadelphia, 1839. Roy. 8vo, pp. 821.

30. Practical Surgery. By Robert Liston, Surgeon. With Notes and additional Illustrations. By G. W. Norris, M.D., one of the Surgeons of the Pennsylvania Hospital.—Phil., 1838. 8vo, pp. 274.

31. Medical and Physiological Problems; being chiefly researches for Correct Principles of Treatment in disputed points of practice. By W. and D. Griffin, M.D.—Limerick, 1839. 8vo, pp. 114.

32. Narrative of the Discoveries of Sir Charles Bell in the Nervous System. By Alex. Shaw, Assist. Surg. to the Middlesex Hospital.—Lond. 1839. 8vo, pp. 252.

33. An Essay on the Functions of Life, comprising demonstrations of a Supreme Inceptive Function in the Organism of Man, New Views, &c. By W. Batten, Esq., Surgeon.—Lond. 1839. 8vo, pp. 36.

34. Documents and Dates of Modern Discoveries in the Nervous System.—London, 1839. 8vo, 5s.

35. A System of Practical Surgery, with numerous Explanatory Plates; the Drawings after nature. By John Lizars, Professor of Surgery to the Royal College of Surgeons, Edinburgh. Part II.—Edinburgh, 1839. 8vo, pp. 333. 24 Plates.

FOREIGN.

1. Das schräg verengte Becken nebst einem Anhange über die wichtigsten Fehler des weiblichen Beckens überhaupt. Von Dr. F. C. Naegele, Professor der Medicin, &c. zu Heidelberg. Mit 16 Tafeln. Mainz, 1839. Folio, pp. 118.

2. Traité des Maladies des Reins, et des Altérations de la Secrétion Urinaire, &c. Par P. Rayer, M.D., &c. Tome I. avec six Planches.—Paris, 1839. 8vo. pp. 638.

3. Die Ermordung der Anna Catharina Höge nach actenmässigen Berichten. Von P. Schmidt, M.D.—Hamb. 1837. 8vo. pp. 212.

4. Die Geburtshülfliche Auscultation. Von Dr. H. F. Naegele.—Mainz, 1838. 8vo. pp. 140.

5. Dissertatio inauguralis de Nervorum regeneratione. Auctore C. O. Steinbrück. (Accedunt duæ Tabulæ.)—Berolini, 1838. 4to. pp. 80.

6. Naschrift op het werkje, over de overeenkomst en het verschil tusschen de Jicht en de Scrophulosis, vooral met betrekking tot de Longtering; door A. A. Sebastian, M.D., &c.—Groningen, 1838. 8vo, pp. 96.

7. De Craniis Esthonum Commentatio Anthropologica. Auctore Alex. Hueck, M.D.—Dorpati Livonorum, 1838. Folio, pp. 16.

8. De Fungo Genu necnon de Tuberculis in hoc Morbo inventis, Dissertatio inaug. quam conscripsit F. J. Lederle.—Petropoli, 1838. 8vo, pp. 81.

9. Viaggio Medico in Germania nella state del 1837. Par B. Bertini, Consigliere del Collegio di Medicina, &c.—Torino, 1838. 8vo, pp. 174.

10. Statistica Nosologica dal 1821 al 1833, e Rendiconto Medico per il 1834, del Venerando Spedale Maggiore, &c. Per Bernardino Bertini, M.D., &c.—Torino, 1835. 8vo, pp. 92.

11. Archivio di Medicina pratica universale compilato dal Dottore A. B. M. Schina, Professore, &c. Sezione Anatomico-fisiologica del Sistema Vasale. Vol. I. II.—Torino, 1836. 8vo, pp. 240, 333.

12. Della Medicina Idiopatica in Germania. Par B. Bertini, M.D.—Torino, 1838. 8vo, pp. 23.

13. Etudes Cliniques sur divers Points de l'Histoire des Fièvres bilieuses et typhoides. Par le Docteur H. C. Lombard, Médecin de l'Hôpital de Genève.—Paris, 1839. 8vo, pp. 40.

14. Lettre sur la Vaccine et les secondes Vaccinations. Par le Dr. H. C. Lombard.—Genève, 1839. 8vo, pp. 50.

15. Memorie della Società Medico-chirurgica di Bologna. Vol. I. Fasc. III. IV.—Bologna, 1837, 1838.

16. Bericht über die Einrichtung und die Ergebnisse der chirurgischen-ophthalmologischen Clinik zu Freiburg während der leztverflossenen neun Jahre, unter der Leitung des verstorbenen Geheimen Hofrathes Dr. Beck. Herausgegeben von Professor Dr. Schwörer.—Freiburg, 1838. 4to, pp. 84.

17. Mémoire sur l'Hémorrhagie des Méninges. Par E. Boudet.—Paris, 1839. 8vo, pp. 64.

LIBRARY OF THE
Orleans Parish Medical Society

### INDEX TO VOL. VIII.

OF THE

### BRITISH AND FOREIGN MEDICAL REVIEW.

PAGE

Abdomen, auscultatory phenomena in 461

Abortion, with retained ovum . 44

Abscess, ileo-cœcal . . . 279

Absorption . . . . . . 311

Ague, Dr. Kremer's theory of . 67

Air-douche in diseases of the ear . 94

Alcohol in the brain . . . 539

Albers, Dr. on fatty disease of the liver 554

Albumen, its relations to urine . 142

Albuminuria, M. Colon on . 121

causes of . . 137

uncertain diagnosis of 249

Alderson, Dr. on colour . . 593

Alison, Professor, his physiology 241

Alum in diseases of the heart . 555

Amputation, a new mode of . 205

spontaneous, case of 567

Anatomy, Wistar's treatise on . 244

Mr. Wilson's practical . 240

Aneurism, treatises on . . 413

thoracic and abdominal 464

Animals, on cruelty to . . 536

Antimony, action of . . . 251

sulphuret of, effects of . 556

Anus, artificial . . . . . 256

Aphonia, cured by ammonia . 252

Apoplexy, cutaneous . . 552

Arnold, Professors, their physiology 476

Arsenic, minimum fatal dose of . 568

new process for detecting 373

Arteries, surgical anatomy of . 538

on the torsion of . . 559

structure and physiology of 171

Arteritis, Dr. Bonetti on . 556

Asphyxia in infants . . 474

Asylum for the insane at Frankford 583

Atavism . . . . . 487

Attention, effects of, on the body . 492

Auscultation, obstetric, Naegele on 365

Barlow, Dr. his address . . 604

Barlow, Dr. on injury of the wrist 588

Beaumont, Mr. on polypi . 237

Beck, Dr. his medical jurisprudence 246

Blasius, Dr. on amputating . 205

on aneurism . 413

Bleeding in brain affections . 489

Blood-vessels in smallpox . 247

Blood, M. Magendie on . . 341

experiments on . 270, 345

state of, after death . 272

its vital properties . 177

viscosity of . . 323

effects of medicine on . 355

PAGE

Blood, state of in plague . 551
Blood-fever . . . 441
Blushing, physiology of . . 245
Bona, in Africa, fevers at . . 57
Bones, the episternal . . 248
Bone formed from periosteum . 285
Books received for review 307, 617
Botany, Mr. Lindley's school . 538
Bouvier, M. on clubfoot . . 384
Bougies, ivory . . . 258
Bowring, Dr. on plague and quarantine 233
Brain, ulceration of . . 275
Brain of the negro, Tiedemann on 374
weight of . . 375, 377
alcohol in . . 539
on bleeding in affections of the 489
Bramley, Mr. on bronchocele . 103
Breschet on the ear . . 68
British Association for Science . 616
Broken head, virtues of a . 557
Bronchocele, its geological relations 106-9
its treatment . 119
treatises on . . 103
Buffy coat, nature of . . 347
Burgess, Dr. on blushing . 245
Buzorini, Dr. on typhus . 428
Cahill, Dr. on delirium tremens . 595
Calculus, pulmonary, large . 279
Calcutta, medical statistics of . 592
Calor mordax, its cause . . 445
Capillaries, physiology of . 175
Carpenter, Dr. on the nervous system 506
Ceely, Mr. on vaccination . 614
Cerebellum, on the functions of . 576
Children, five at a birth . 564
Chlorosis, Mr. Fox on . . 542
with menorrhagia . 250
Blaud's pills in . 252
Christison, Dr. on disease of kidneys 121
Churchill, Dr. on version . 597
Chyle, its progress into blood . 479
Circocele, operation for . . 558
Circulation, pulmonary . . 329
Claubry, M. on typhus fever . 428
Clanny, Dr. on Wourali poison . 597
Club foot, treatises on . . 384
causes of . . 388
operation for . . 391
Cod-liver oil in scrofula . . 554
Colchicum, on the use of . . 499
Colon, on the pathology of . 501
Colour of animals, certain causes of 593
Colchicum, external use of . 280

| | PAGE |
|---|---|
| Coudret, M. on animal electricity | 406 |
| Congestion of blood, nature of . | 182 |
| Consumption in West Indies . | 219 |
| Contagion, mode of propagating | 315 |
| Convulsions, singular case of . | 595 |
| Cooper, Sir A. anecdote of . | 535 |
| Copaiba, rheumatism from . | 280 |
| Cowpox, identity of, with smallpox | 614 |
| Craig, Mr. on protracted labours . | 467 |
| Cupro-ammoniacal liquor . | 353 |
| Cutaneous diseases, Dr. Willis on . . . | 239-541 |
| Davidson, Mr. on the perinæum . | 284 |
| Davies, Mr. on iodine . . | 512 |
| Davy, Sir H. his Life . . | 545 |
| Dr. on the blood and respiration | 269 |
| his life of Sir H. Davy . | 545 |
| De Candolle, M. his organography | 243 |
| Deleau, M. on the ear . . | 68 |
| Delirium tremens, on opium in . | 593 |
| Determination of blood . . | 181 |
| Dewar, M. on convulsive diseases | 595 |
| Dickson, Dr. his unity of disease | 531 |
| Dierbach, Dr. on materia medica . | 353 |
| Digestion, experiments on . | 478 |
| Diseases, on the connexion of . | 501 |
| Druitt, Mr. his vade mecum . | 536 |
| Dunglison, Dr. his medical library | 539 |
| on the temp. of the uterus | 591 |
| his medical lexicon . | 548 |
| on the fœtal heart . | 581 |
| Dunn, Mr. on dislocation of femur | 286 |
| Dysmenorrhœa, Dr. Meigs on . | 40 |
| Ear, treatises on . . | 68 |
| Education, medical . . | 287 |
| Elasticity . . . | 326 |
| Electricity, animal . . | 406 |
| its relation to fever . | 445 |
| Electromotor, a new medical agent | 409 |
| Ellis, Mr. on hernia . . | 596 |
| Endosmosis and exosmosis . | 317 |
| Exploration, uterine, importance of | 370 |
| Eustachian tube, anatomy of . | 75 |
| catheterism of . | 93 |
| Evidence, medical, nature of . | 483 |
| Evolution, spontaneous, case of . | 567 |
| Excito-motory system . . | 508 |
| Exhalation . . . | 317 |
| Eye, anatomy of . . . | 71 |
| physiology of . . | 82 |
| diseases of . . . | 89 |
| Eye, development of hair in . | 256 |
| Morgan on diseases of . | 329 |
| Face-presentation, causes of . | 258 |
| Family diseases not hereditary . | 287 |
| Farcy in the human subject . | 552 |
| Femur, congenital dislocation of . | 559 |
| Fever, typhus, treatises on . | 428 |
| use of wine in . . | 281 |
| intermittent, ligature in . | 251 |
| narcotine in | 263 |
| irritative, from hot liquids . | 277 |
| yellow, its fatality . | 214 |
| Finger, permanent flexure of . | 401 |
| Flood, Dr. on the arteries . . | 538 |
| Fœtus, on the pulsations in . | 581 |
| origin of hernia in . | 594 |
| Food, Dr. Holland, on deficiency of | 524 |
| deficiency of, symptoms of . | 525 |
| treatment of . | 526 |
| Fourchette, laceration of . . | 38 |
| Fox, Mr. on chlorosis . . | 542 |
| Fractures, statistics of . . | 592 |
| Galvanic midwifery forceps . | 666 |
| Gangrene, from tight bandaging . | 558 |
| Gases permeate tissues . . | 322 |
| Gastrodynia, treatment of . . | 358 |
| Gonorrhœa, secondary symptoms of . . . | 426 |
| Gout and scrofula, relation of . | 242 |
| nature of . | 497 |
| Groin, surgical anatomy of . . | 542 |
| Guerin, on section of spinal muscles | 557 |
| Hair developed in the eye . . | 256 |
| Halliday, on West India diseases . | 211 |
| Hanging, a new sign of . . | 572 |
| Heart, state of, in fever . . | 281 |
| wound of . . . | 255 |
| Dr. Hope on diseases of . | 448 |
| natural sounds of . . | 448 |
| on alum in diseases of . | 555 |
| Heart, fœtal, on the pulsations of . | 581 |
| Henderson, Mr. on sea-scurvy . | 595 |
| Hepatitis, mercurial inunction in . | 420 |
| Hernia, on trusses in . . | 561 |
| Hereditary disease . . . | 484 |
| Hermaphrodism, M. St. Hilaire on | 1 |
| Hernia, Dr. O'Beirne on . . | 596 |
| Hilaire, St. M. on monstrosities . | 1 |
| Hemorrhage, uterine, treatment . | 475 |
| Holland, Dr. his medical notes . | 482 |
| Dr. B. on food . | 524 |
| Homœopathic doctrines . . | 427 |
| Hope, Dr. on diseases of the heart . | 448 |
| Hope, Dr. on the spleen . . | 393 |
| Hosack, Dr. on tumours of the urethra . . . | 585 |
| Hunter, John, on inflammation . | 169 |
| animal magnetism | 493 |
| Hydriodate of potash, preparation of | 597 |
| Hydrocephalus, acute, mercury . | 426 |
| on compression in . | 560 |
| Hydrocyanic acid in gastrodynia . | 358 |
| Hydrostatics, vital . . | 528 |
| Imbibition, physiological . . | 311 |
| Inflammation, Hunter's theory of . | 183 |
| Macartney's ditto | 184, 186 |
| Dr. Carswell's ditto . | 189 |
| Mr. Davies on . | 573 |
| anatomical characters of | 193 |
| treatment of . . | 198 |
| treatises on . . | 169 |
| Magendie on . | 339 |
| Influenza, an epidemic . . | 502 |
| Inglis, Dr. on bronchocele . | 103 |
| Iris, on the physiology of . . | 580 |

2 3

PAGE
Insanity, American treatment of . 583
Insect life, as a cause of disease . 506
Intermittence, nature of . . 505
Intermittent inflammations . . 421
fever, treatises on . 56
Introsusception, mercurius vivus in 422
Iodine, Mr. Davies on the use of, 512-18
diseases in which it is useful 521
its use in herpes . . 591
Iron, sesquioxyde, preparation of . 360
antidote of arsenic 573
Irrigation in inflammation . . 200
Jackson, Dr. on typhus fever . 428
Jahn, Dr. on practical medicine . 419
Jaw, lower, peculiar disease of . 423
Jeffreys, Dr. his address . . 605
Jones, Mr. on the diseases of women 529
Jurisprudence, medical, elements of 246
Kennedy, Dr. on prolapsus uteri . 596
Kidneys, treatises on diseases of . 121
table of diseases of . 125
Knee-joint, contracted, operation for 400
Köchlin, Dr. on metallic medicines 353
Labours, protracted, treatise on . 467
treatment of . 471
Laycock, Mr. on colchicum . 280
Lee, Dr. his physiology for schools 533
Lens, on the reproduction of . . 247
Leucorrhœa, Dr. Meigs on . 41
Life, physical phenomena of . 309
Liquor amnii, morbid accumulation of 564
Lime, on the use of . . 359
Lincke, Dr. on the ear . . 68
Lindley, Mr. his school botany . 538
Little, Dr. on club-foot . . 384
Liver, on fatty disease of . 554
Lombard, Dr. on typhus . . 428
Longevity in Prussia . . 575
Löwenhardt, L. on practical medicine 419
Macaulay, on cruelty to animals . 536
M'Clelland, Mr. on bronchocele . 103
Maddock, Dr. on copaibal rheumatism 280
Magendie, M. on life . . 309
Magnetism, effects of . . 481
animal . . 494
Maillot, M. on intermittent fevers 56
Malformation, case of . . 566
Malgaigne, M. on hernia . 561
Marshall, Mr. on soldiers . 537
Martin, M. on pregnancy . 37, 50
Materia medica, treatises on . 353
Maunsell, Dr. on political medicine 547
May, Dr. on ulceration of the brain 275
Membrana tympani, anatomy of . 73
Meigs, Dr. his treatise on midwifery 37
Menstruation . . . 39
theory of . . 530
Mercurial inunctions, large, use of 419
Metals, their medical use . . 353
Metallic tinkling . . . 451
Middle ear, diseases of . . 99
Midwifery, treatises on . 37, 50, 467

PAGE
Mitscherlich, Dr. on materia medica 353
Mollusca, on the nervous system of 507
Monsters, different kinds of . 29
Monstrosities, St. Hilaire on . 1
classification of . 4, 18
general laws of 4, 7, 8, 11
Montault, M. on typhus . . 428
Moon, its influence on disease . 481
Morphia, bimeconate of . . 275
Morgan, Mr. on diseases of the eye 229
Mortality in Prussia . . 578
Morton, on the perineum . . 244
on the groin . . 542
Naegele on obstetric auscultation 365
Narcotine in intermittent fever . 263
Nasmyth on the teeth . . 159
Negro, comparative size of brain of 374
Nephritis, varieties of . . 150
diagnosis of . . 152
treatment of . . 156
Nerves, functions of the eighth pair 264
Nerves, on the lesion of ganglionic 594
Nervous system, anatomy of . 226
Nervous system of the invertebrata 506
Newnham, Mr. his surgical report 546
Nicholls, Mr. on hydriodate of potass 597
Notes and reflections, Dr. Holland's 482
Nutrition, physiology of . . 549
O'Beirne, Dr. on hernia . . 596
Old age, medical treatment of . 503
Ophthalmia, conium in . . 256
Opium, its use in fever . . 439
Organography, vegetable . . 243
O'Shaughnessy, Dr. on narcotine . 263
Otitis . . . . . . 91
Otto, Dr. on cutaneous apoplexy . 552
Paget, Mr. on blood-vessels in tendons 594
Palsy, treatment of . . . 490
Pathology, Dr. Arnold's . . 476
Patient, when he may judge for himself 495
Pelvis, diameter of . . . 37
Percy, Dr. on alcohol in the brain . 539
Pericarditis . . . . 463
Pereira, Mr. his Materia Medica . 353
Perineum, surgical anatomy of . 244
laceration of . . 284
Periosteum, forms bone . . 245
Perspiration, effects of . . . 491
Permeability of tissues, physiological 322
Phillips, Mr. on prolapsus uteri . 596
on division of tendons ib.
Pilcher, Mr. on the ear . . 68
Phthisis, given to animals . . 249
Physiology, Dr. Arnold's . . 476
Dr. Alison's . . 241
Physiology for schools . . 533
Pirondi, Dr. his travels in England 534
Placenta, natural position of . 371
Plague, observations on . . 233
morbid anatomy of . 550
Poisons, mode of operation of . 314
Poisoning by sulpho-cyanic acid . 258

PAGE
Political medicine, Dr. Maunsell on . 547
Polypi, ligature of . . 237
Porosity, physiological . . 311
Pregnancy, diseases complicating . 50
Prolapsus uteri, new treatment of 596
Provincial Med. Association . 303, 603
Puerperal fever . . 49
Pulse, variability of in the infant . 367
in diseases of the heart 456-466
Purgatives, on the abuse of . 496
Quackery, report on . . 610
Quarantines, Dr. Bowring on . 233
Rash, the typhoid . . 432-436
Rayer, M. on the kidneys . 121
Reflections and Notes, Dr. Holland's 482
Reform, medical . . . 287
report on . . 612
petition for . . 610
Registration of births and deaths . 532
Reid, Dr. on the eighth pair of nerves 264
on the ganglionic nerves 594
Revaccination in the Prussian army 261
Mr. Newnham on . 546
Roupell, Dr. on typhus . . 428
Rumsey, Dr. on irritative fever . 277
Sawkins, Mr. on hernia . . 596
Scrofula and gout, relation of . 242
Scurvy, Mr. Henderson on . 595
Sebastian, Dr. on gout and scrofula 242
Simon, Dr. on nutrition . . 549
Simpson, Dr. on fœtal pathology . 594
Smallpox, anatomy of . . 247
identity of with cowpox 614
Snake, living swallowed . . 257
Societies, medical, English . 535
Soldiers, on enlisting . . 537
Solon, M. on albuminuria . 121
Somervail, Dr. on tobacco . 582
Speculum, Mr. Jones on the . 529
Spinal marrow, respiratory tract of 480
Spinal muscles, section of . . 557
Spine, tenderness of, in ague . 67
supposed diseases of . 500
Spleen, rupture of . . 248
structure and functions of . 593
Squire, Mr. on morphia . . 275
Stillborn children, restoration of 596
Stokes, Dr. on wine in fever . 281
Stromeyer, Dr. on orthopœdia . 384
Sudorific medicines . . 490

PAGE
Surgical literature, report of . 546
Swan, Mr. on the nervous system 226
Syme, Mr. on the formation of bone 285
Teeth, treatises on the structure of 158
development of . . 165
Tendons, on the division of . 384
Tendon achilles, section of the . 385
Tendons, on the circulation in . 594
hamstring section of . 596
Teratology, St. Hilaire on . 1
Terry, Mr. on suspended animation 596
Tobacco, on the external use of . 582
Todd, Dr. on the ear . . 68
Tracheotomy, statistics of . 558
Trismus in Iceland . . 486
Troops, British, on the health of . 211
Tulloch, Major, on the army . ib.
Twins united, case of . . 568
Tympanum, anatomy of . . 76
Typhus fever, treatises on . . 428
contagiousness of . 430
treatment of 438-447
Typhoseptoses, a family of diseases 442
Unity of disease . . 531
University of London, regulations of 287
examiners in 304
Urethra, female, on tumours in . 585
Urine, chemistry and pathology of 126
critical . . . 157
Vaccination, experiments in . 614
petition respecting . 613
Vade mecum, Mr. Druitt's . 536
Vagina, muscularity of . . 39
Vagina and uterus, temperature of 591
Valves, diseased, without murmur . 279
Valves, diagnosis of . . 452
Varicocele, case of . . 253
Veins, wounds in . . 460
Veratrine, researches on . 353
effects of . . 363
Version, obstetrical, statistics of . 597
Wardrop, Dr. on aneurism . 413
Waterton, Mr. on Wourali poison 597
Willis, Dr. on cutaneous diseases 239-541
Wilson, Mr. his surgical anatomy . 240
Women, on the diseases of . 529
Wormald, his Anatomical Sketches . 542
Wourali poison, experiments with . 597
Wrist, peculiar injury of . . 588
Wry-neck, case of . . 402

END OF VOL. VIII.

PRINTED BY C. ADLARD, BARTHOLOMEW CLOSE.

